# Malignant struma ovarii with papillary thyroid carcinoma: a case report

**DOI:** 10.1093/jscr/rjag264

**Published:** 2026-04-15

**Authors:** Rim Laaboudi, Nassima Ouguerzi, Hounaida Mahfoud, Fatima El Hassouni

**Affiliations:** Gynaecology-Obstetrics, Cancerology and High-Risk Pregnancy-Maternity Souissi, University Hospital Center, Avenue Ibn Sina, Souissi, Rabat 10112, Morocco; Gynecology-Obstetrics and Endocrinology-Maternity Souissi, University Hospital Center, Avenue Ibn Sina, Souissi, Rabat 10112, Morocco; Gynecology-Obstetrics and Endocrinology-Maternity Souissi, University Hospital Center, Avenue Ibn Sina, Souissi, Rabat 10112, Morocco; Gynaecology-Obstetrics, Cancerology and High-Risk Pregnancy-Maternity Souissi, University Hospital Center, Avenue Ibn Sina, Souissi, Rabat 10112, Morocco

**Keywords:** struma ovarii, papillary thyroid carcinoma, ovarian teratoma, monodermal teratoma, ectopic thyroid tissue, malignant struma ovarii

## Abstract

Struma ovarii is a rare monodermal ovarian teratoma predominantly composed of thyroid tissue, accounting for <5% of mature ovarian teratomas. Malignant transformation, most commonly to papillary thyroid carcinoma, occurs in 5%–10% of cases and represents an exceptionally rare entity with no established management consensus. We report a 40-year-old woman presenting with acute pelvic pain and a 13 cm heterogeneous left adnexal mass with elevated CA-125 (125 U/ml). Emergency laparotomy with left salpingo-oophorectomy revealed a 1 cm papillary thyroid carcinoma arising within an 11 cm struma ovarii, without capsular or vascular invasion. Postoperative thyroid workup was normal. Despite multidisciplinary recommendations for completion thyroidectomy and contralateral adnexectomy, the patient declined further surgery, opting for clinical and biochemical surveillance alone. This case underscores the importance of managing malignant struma ovarii according to thyroid carcinoma protocols while respecting patient autonomy.

## Introduction

Struma ovarii, first described by Von Kalden in 1895, is an uncommon monodermal variant of mature cystic teratoma defined by the predominance of thyroid tissue constituting more than 50% of the tumour volume [1]. This entity accounts for ~2.7% of all ovarian teratomas and predominantly affects premenopausal women, with 85% of reported cases occurring in this demographic [2]. Although struma ovarii follows a benign course in the vast majority of cases, malignant transformation is documented in 5%–10% of instances, with papillary thyroid carcinoma (PTC) representing the most frequently encountered histological subtype [3].

The clinical presentation of struma ovarii is characteristically nonspecific, closely mimicking that of other ovarian neoplasms. Affected patients typically present with pelvic pain, a palpable abdominal mass, or menstrual irregularities. In a minority of cases, autonomous ectopic thyroid hormone secretion may give rise to clinical hyperthyroidism, a phenomenon reported in 5%–8% of patients [4]. While radiological findings may be suggestive, they are not pathognomonic, and definitive diagnosis invariably relies upon histopathological examination. Owing to the extreme rarity of malignant struma ovarii, no consensus has been established regarding optimal therapeutic strategies, particularly with respect to the extent of primary surgery, the role of completion thyroidectomy, and the indications for adjuvant radioactive iodine therapy.

We report a case of PTC arising within struma ovarii in a 40-year-old woman presenting with acute pelvic pain, who subsequently declined the recommended surgical management—underscoring the clinical and ethical challenges inherent in treating this rare malignancy when patients exercise their right to autonomous decision-making.

## Case report

A 40-year-old woman, gravida 2 para 2, presented to the gynaecological emergency department with acute pelvic pain localized to the left iliac fossa. Her past medical history was unremarkable, with no prior thyroid disorder or ovarian pathology.

Physical examination revealed a firm, non-mobile, tender pelvic mass of ~13 cm on bimanual examination, with associated abdominal guarding. The patient was clinically euthyroid, with no signs of thyrotoxicosis. Vital signs were stable.

Emergency abdominopelvic ultrasound demonstrated a large heterogeneous left adnexal mass with mixed solid and cystic components measuring 13 cm. Colour Doppler revealed partially chaotic internal vascularization. According to the International Ovarian Tumour Analysis criteria, these features were highly suspicious for malignancy. The contralateral adnexa and uterus appeared sonographically normal, with no ascites or lymphadenopathy identified. Given the clinical suspicion of adnexal torsion, cross-sectional imaging was not performed prior to surgery.

Laboratory investigations revealed moderate anaemia (haemoglobin 8.5 g/dl) and a markedly elevated CA-125 of 125 U/ml (reference range < 35 U/ml). Thyroid function tests were deferred in the acute setting.

**Figure 1:**
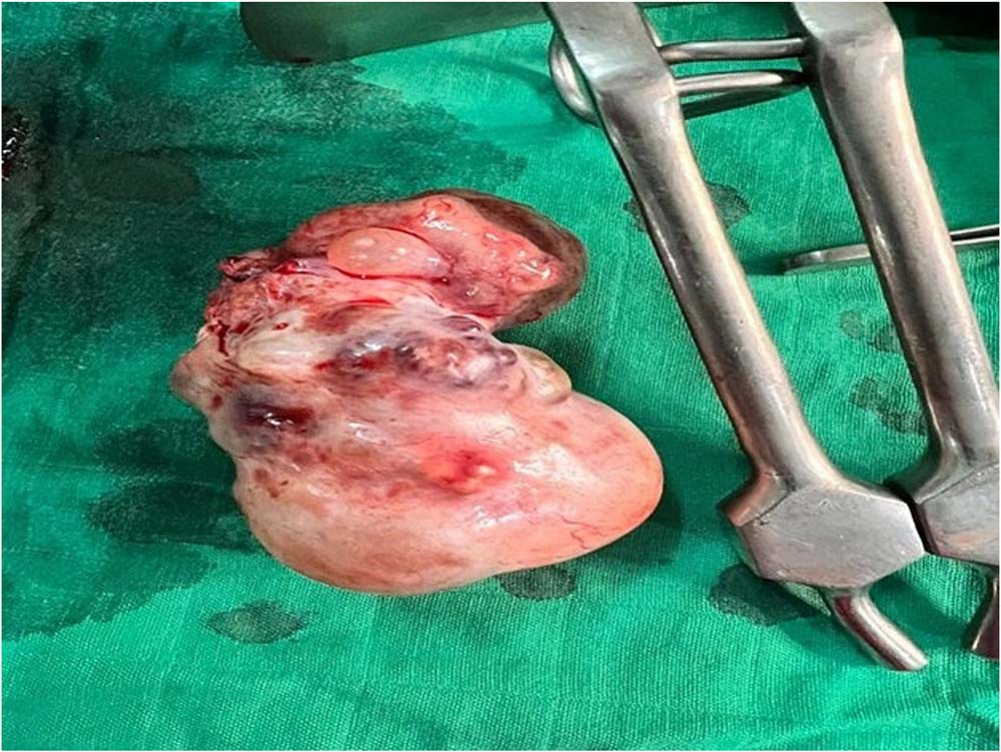
Surgical specimen following left salpingo-oophorectomy, demonstrating a polylobulated solid ovarian mass with intact capsule and areas of haemorrhagic change on the external surface.

The patient underwent emergency exploratory laparotomy. Intraoperative findings disclosed a large solid ovarian mass arising from the left ovary, measuring 20 cm in its greatest diameter, with an intact capsule and no peritoneal implants or evidence of metastatic disease. A left salpingo-oophorectomy was performed without complication (Fig. 1).

**Figure 2:**
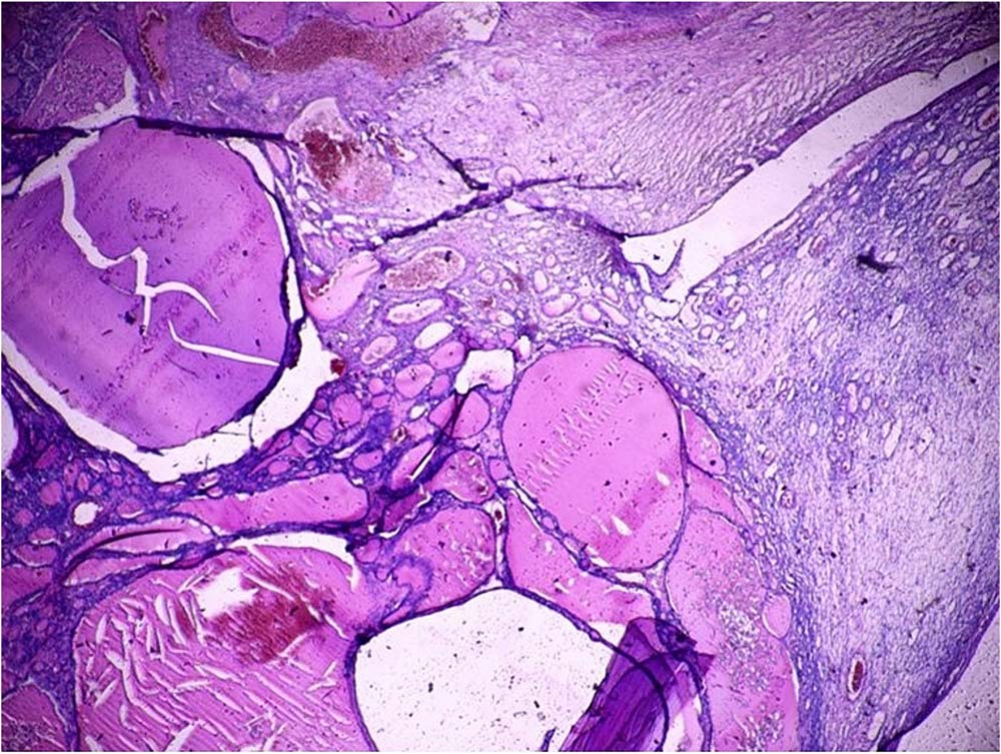
Histopathological section (H&E, ×40) demonstrating thyroid follicles of varying sizes filled with colloid material, consistent with struma ovarii comprising more than 50% of the ovarian tumour volume.

**Figure 3:**
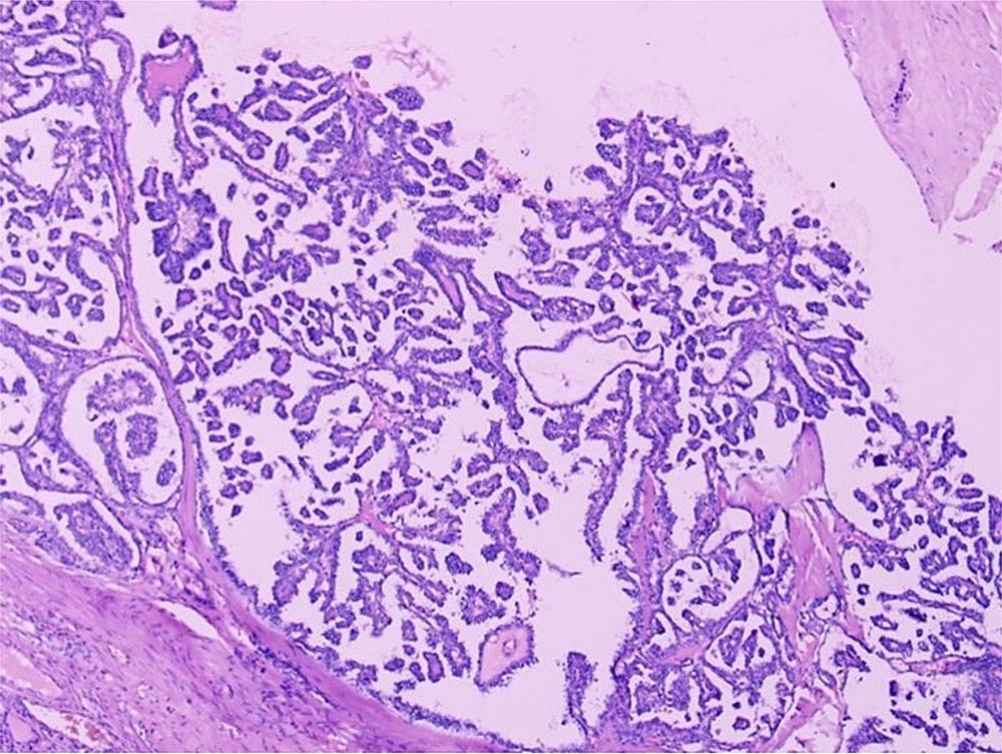
Histopathological section (H&E, ×100) demonstrating papillary thyroid carcinoma arising within struma ovarii, characterized by complex papillary fronds with fibrovascular cores lined by neoplastic cells exhibiting crowded overlapping nuclei. The adjacent benign thyroid follicles are visible on the left.

Histopathological examination confirmed a diagnosis of struma ovarii measuring 11 cm, within which a 1 cm focus of PTC was identified (Figs 2 and 3), exhibiting the characteristic nuclear features of PTC. No capsular or lymphovascular invasion was demonstrated, and the tumour was classified as Stage IA. Postoperative thyroid evaluation, including ultrasonography and thyroid function tests, was entirely normal.

Following multidisciplinary tumour board review, a management plan was formulated comprising completion total thyroidectomy, total abdominal hysterectomy with right salpingo-oophorectomy, and long-term surveillance with serial thyroglobulin measurements. After thorough counselling regarding the oncological rationale and the risks of non-treatment, the patient declined all further surgical intervention, citing personal reasons whilst demonstrating clear understanding of the recommendations. She consented to clinical and biochemical surveillance alone, comprising clinical examination every 3–6 months, thyroid function tests and serum thyroglobulin measurement every 6 months, and annual pelvic ultrasonography and chest radiography. The absence of thyroidectomy significantly limits the sensitivity of thyroglobulin monitoring and precludes the use of radioactive iodine therapy.

## Discussion

This case illustrates the diagnostic, therapeutic, and ethical challenges inherent in the management of malignant struma ovarii, a rare entity for which no standardized treatment consensus exists.

Struma ovarii is classified as a monodermal teratoma derived from endodermal germ cells that undergo preferential differentiation towards thyroid tissue. Malignant transformation occurs in 5%–10% of cases, with PTC accounting for ~80% of malignant cases [1, 3]. The biological behaviour of these tumours closely parallels that of primary thyroid malignancies, and management should accordingly follow thyroid carcinoma protocols rather than ovarian cancer paradigms [5].

The clinical presentation is characteristically nonspecific. Pelvic pain, the predominant symptom in our patient, is reported in ~12% of cases, while a palpable abdominal mass is the most common presentation, occurring in up to 58% [4]. Clinical hyperthyroidism, though reported in 5%–8% of patients due to autonomous ectopic hormone production, was absent in our case despite the substantial thyroid tissue volume—a finding consistent with the majority of published series [3].

Preoperative diagnosis of struma ovarii remains challenging, as no serum biomarker is pathognomonic for this entity. CA-125 elevation, as observed in our patient, is nonspecific. Serum thyroglobulin may serve as a useful tumour marker following thyroidectomy, when native thyroid gland contribution is eliminated [6]. In our case, the absence of baseline thyroglobulin measurement and the patient's subsequent refusal of thyroidectomy significantly limit surveillance efficacy.

Total thyroidectomy is strongly recommended in malignant struma ovarii for three principal reasons: to exclude a synchronous primary thyroid malignancy, to enable radioactive iodine ablation of any residual or metastatic thyroid tissue, and to optimize thyroglobulin-based surveillance [7]. In our patient, the refusal of completion thyroidectomy precludes both radioactive iodine therapy and sensitive tumour marker monitoring, representing a significant limitation in long-term oncological follow-up.

With respect to surgical staging, the absence of formal peritoneal washings, omental biopsy, and lymph node assessment during the initial emergency procedure represents an acknowledged limitation. Intraoperative exploration revealed no macroscopic evidence of metastatic disease; nonetheless, microscopic dissemination cannot be definitively excluded.

The prognosis of localized papillary carcinoma within struma ovarii is generally favourable, with reported 10-year survival rates exceeding 90% [1, 3]. Our patient's pathological profile—a 1 cm focus of PTC with no capsular or lymphovascular invasion and no extraovarian extension—is associated with an excellent prognosis. Nevertheless, the long-term impact of incomplete surgical management remains uncertain.

Finally, this case raises important ethical considerations regarding patient autonomy. Following thorough and documented counselling, the patient exercised her right to decline recommended surgical intervention. Respecting informed refusal while maintaining close surveillance and preserving the therapeutic alliance represents the cornerstone of ethical oncological practice in such challenging circumstances.
